# What lies underneath: Precise classification of brain states using time-dependent topological structure of dynamics

**DOI:** 10.1371/journal.pcbi.1010412

**Published:** 2022-09-06

**Authors:** Fernando Soler-Toscano, Javier A. Galadí, Anira Escrichs, Yonatan Sanz Perl, Ane López-González, Jacobo D. Sitt, Jitka Annen, Olivia Gosseries, Aurore Thibaut, Rajanikant Panda, Francisco J. Esteban, Steven Laureys, Morten L. Kringelbach, José A. Langa, Gustavo Deco

**Affiliations:** 1 Grupo de Lógica, Lenguaje e Información, Universidad de Sevilla, Seville, Spain; 2 Departamento de Ecuaciones Diferenciales y Análisis Numérico, Universidad de Sevilla, Seville, Spain; 3 Computational Neuroscience Group, Center for Brain and Cognition, Universitat Pompeu Fabra, Barcelona, Catalonia, Spain; 4 Institut du Cerveau et de la Moelle épinière, ICM Paris, Paris, France; 5 Universidad de San Andrés, Buenos Aires, Argentina; 6 Inserm U 1127, Paris, France; 7 CNRS UMR 7225, Paris, France; 8 Coma Science Group, GIGA-Consciousness, Liège University, Liège, Belgium; 9 Centre du Cerveau^2^, University Hospital of Liège, Liège, Belgium; 10 Departamento de Biología Experimental, Universidad de Jaén, Jaén, Spain; 11 Centre for Eudaimonia and Human Flourishing, Linacre College, University of Oxford, Oxford, United Kingdom; 12 Department of Psychiatry, University of Oxford, Oxford, United Kingdom; 13 Center for Music in the Brain, Department of Clinical Medicine, Aarhus University, Aarhus, Denmark; 14 Institució Catalana de la Recerca i Estudis Avançats (ICREA), Universitat Pompeu Fabra, Barcelona, Spain; Yale, UNITED STATES

## Abstract

The self-organising global dynamics underlying brain states emerge from complex recursive nonlinear interactions between interconnected brain regions. Until now, most efforts of capturing the causal mechanistic generating principles have supposed underlying stationarity, being unable to describe the non-stationarity of brain dynamics, i.e. time-dependent changes. Here, we present a novel framework able to characterise brain states with high specificity, precisely by modelling the time-dependent dynamics. Through describing a topological structure associated to the brain state at each moment in time (its attractor or ‘information structure’), we are able to classify different brain states by using the statistics across time of these structures hitherto hidden in the neuroimaging dynamics. Proving the strong potential of this framework, we were able to classify resting-state BOLD fMRI signals from two classes of post-comatose patients (minimally conscious state and unresponsive wakefulness syndrome) compared with healthy controls with very high precision.

## Author summary

Brain states emerge through continuously evolving dynamics of brain networks. The usual way of modelling these dynamics is by using stationary systems: there is one structure (attractor) which is responsible of the brain dynamics. We adopt a different approach by characterising the brain activity through a landscape of informational structures (IS) changing in time. We use a model transformation procedure to produce these structures and look at several properties related to how the different brain networks interact not in the observed resting-state fMRI signal but in the information structure underlying it. These properties provide measures strongly related with relevant characteristics of conscious activity, such as metastability, information integration or synchronisation. The distribution of IS measures is studied for healthy controls (HC) and two groups of post-comatose patients with disorders of consciousness (DOC): minimally conscious state (MCS) and unresponsive wakefulness syndrome (UWS). Based on IS measures, machine learners classifiers identify the state of consciousness with an outstanding discrimination (precision of 95.6% por HC/DOC and 86.6% for MCS/UWS).

## Introduction

Brain states such as wakefulness, sleep, and altered states of consciousness emerge through continuously evolving dynamics of self-organised whole-brain networks which, depending on the underlying condition, go through transient metastable arrangements [1]. The challenging question is how best to describe them through disentangling the mechanisms underlying functional neuroimaging data capturing their whole-brain dynamics over time and space [2–5]. Whole-brain models have been very successful in capturing many of the key features of brain dynamics including spatiotemporal aspects [2, 6]. But they assume that the underlying energy landscape (the function providing the direction of change for each point in the phase space, i.e., the repertoire of states of the system) is stationary (that is, it does not change across time), which is not a realistic assumption in most cases [7, 8]. In general, until now, it has proven very difficult to fully describe the principles generating the brain dynamics.

Here, the aim is to develop a mathematical formal framework able to characterise the dynamics of brain states with high specificity. The mathematical field of Dynamical Systems Theory studies coupled-systems of differential equations in which the behaviour of each variable depends on the others by a given function. In the Lotka-Volterra (LV) systems used in this paper, the dynamics of each component (a brain region) depends on the other components and the corresponding parameters. It has been shown that each of these systems has an associated global attractor [9, 10], which is a set including all points that represent stationary configurations of the system and all their connections. Thus, there is a structure linking all these stationary points, associated to trajectories going from one stationary point to another one. The stability and instability directions of each stationary point are characterised by non-stationary solutions entering or leaving these points, respectively. A distinguished stationary point is the globally asymptotically stable solution (GASS) which is maximally stable (only stability directions). We call Information Structure (IS) the graph containing all stationary points and the trajectories connecting them [11]. Under some conditions, all stationary points in the IS can be ordered by their level of attraction or stability (energy levels [12]).

The Attractor Landscape (AL) analysis converts the behaviour of a dynamical system into a landscape of stationary points towards which the system potentially tends to approach or move away. Every state of the system at a particular time is affected by the AL (the “information” of the IS is because of its ability to influence every point in the phase space). This provides a number of topological measures in the structure of this attractor landscape, namely NoEL (number of energy levels in the IS), frondosity (number of stationary nodes), criticality (sensitivity to perturbations), synchronicity (probability of all brain networks to behave in the same way) and cooperation (how some networks are influenced by others). More details about IS measures can be found in Methods. The IS can be computed exactly when the number of dynamical variables is low by a Lotka-Volterra Transform (LVT) procedure [7, 13]. This simple model matches the conditions so that the invariants can be ordered in energy levels, supports an algorithm of low computational complexity, and has well-known conditions for the existence and uniqueness of its global attractor, which can be characterized in detail.

Here, we apply this precise mathematical framework to two empirical neuroimaging datasets (Liège and Paris) including BOLD fMRI resting state signals of healthy controls (HC) and two groups of post-comatose patients with disorders of consciousness (DOC) after severe brain injury: minimally conscious state (MCS) and unresponsive wakefulness syndrome (UWS), also known as vegetative state. Patients in MCS [14] present some behaviour that may indicate awareness (i.e., visual pursuit, orientation to pain, or reproducible command following). Patients in UWS [15] show arousal (eyes opening) but unresponsive (no response to simple commands) and without signs of awareness (never presenting non-reflex voluntary movements). Yeo’s parcellation [16] into seven brain networks and the use of LVT provide the parameters of LV equations allowing the characterisation of the instantaneous IS of the brain at each moment in time. This IS can be studied through several measures. Repeating this for all time points in the data provides statistics (i.e. the mean and variability of the IS measures) capturing a brain state accurately, characterised by the exact non-stationary underlying “information structures” hitherto hidden in the neuroimaging dynamics. Thanks to the Dynamical Systems Theory we know that the fundamental information of a dynamical system is expressed in the structure of its global attractor. Thus, the aim of this paper it to test the hypothesis that, in the case of the human brain as a dynamical system, the structure of the attractor could be related to the corresponding states of consciousness.

## Methods

### Ethics statement

This study was conducted according to the Helsinki Declaration. Written informed consent for participation in the study was obtained from healthy controls and the patients’ legal surrogates. Study protocols were approved by the Ethics Committee of the Faculty of Medicine of the University of Liège and the Comite de Protection des Personnes Ile de France 1.

**Fig 1:**
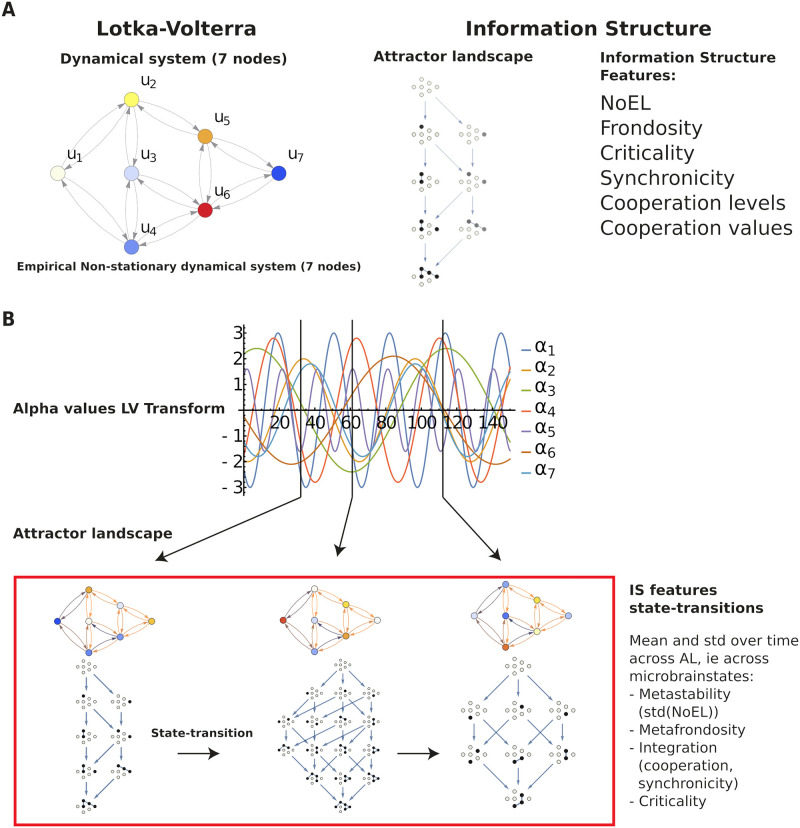
Information structures and their dynamics. **A**. Left: Lotka-Volterra 7-node dynamical system. Relevant parameters are those affecting just one node (*α*_*i*_) and those representing interactions, given by the weighted links (*γ*_*ij*_) between nodes (*i* and *j*). Right: Associated to each system (for given *α*_*i*_ and *γ*_*ij*_ parameters) there is an Information Structure (IS), containing the attractor landscape, that is, stationary points of the system dynamics and the solutions linking them. Black nodes in the stationary points represent a value greater than 0 of the corresponding node of the dynamical system. White nodes stand for 0 value of the corresponding node. Different *γ*_*ij*_ and *α*_*i*_ values produce different IS in both the globally asymptotically stable solution (GASS, in the bottom) and the nodes in the intermediate levels. The structure of the IS can be studied through several measures (see Fig 2) depending not only on the number of levels and nodes (NoEL and Frondosity, respectively) but also on the interaction between nodes (Synchronicity, Criticality and Cooperation measures). **B**. For a dynamical system with fixed connectivity (*γ*_*ij*_ parameters) and changing activity among the nodes (*α*_*i*_) it is possible to study the evolution of the IS measures. The top graphic represents the change in time of the *α*_*i*_ parameters in a Lotka-Volterra 7-node system with fixed connectivity *γ*_*ij*_. Associated to each instant there is an IS with the structure of the attractor underlying the dynamics of the system. The evolution of the system dynamics can be characterised by looking at the mean and standard deviation across time of the IS measures.

We aim to characterise different consciousness states by looking at the evolution in time of the attractor underlying the brain dynamics (Fig 1A). Lotka-Volterra (LV) cooperative systems are used, in which the equation modelling the dynamics of each node *u*_*i*_ depends on a parameter *α*_*i*_ intrinsic to the given node and parameters *γ*_*ij*_ modelling the influence on *u*_*i*_ of each node *u*_*j*_. The shape of the equations is $u_{i}^{\prime }=u_{i}\left(\alpha_{i}-u_{i}+\sum_{j\neq i}^{n}\gamma_{ij}u_{j}\right)$ for *i* = 1, …, *n*. Each *u*_*i*_ represents the timeseries of the BOLD fMRI signal mapped to each of the brain networks in the Yeo’s 7-network parcellation atlas: (1) Visual, (2) Somatomotor, (3) Dorsal attention, (4) Ventral attention, (5) Limbic, (6) Frontoparietal, and (7) Default Mode Network [16].

To extract the Information Structure at each instantaneous time point, we apply the Lotka-Volterra Transform (LVT) [7, 13]. Similar to whole-brain models, this consists of coupling local dynamics with anatomical brain connectivity. The input of LVT are the *u*_*i*_ timeseries and the structural connectivity (SC) matrix containing all *γ*_*ij*_ parameters. The matrix used for all participants was the average SC matrix of all Liège HC subjects, given the unavailability of the connectivity matrix of many of the DOC patients. Using a different matrix for each group would have introduced differences that are not due to brain activity but to the different SC matrices. The output of LVT is the evolution in time of the *α*_*i*_ parameters which reproduce the BOLD fMRI signals. The application of LVT requires two coupling parameters. The first of them, *h*, is used to discretize the LV equations. The second one, *g*, multiplies the SC matrix in order to weight the relevance of connectivity with respect to the *α* values. For the data acquired with TR = 2000 ms (all participants from Liège and 32 from Paris: 21 MCS and 11 UWS), *h* = 3.55 × 10^−4^ was used in the LVT. For data with TR = 2400 ms. (13 HC, 11 MCS and 10 UWS in the Paris group) *h* = 4.26 × 10^−4^ was used (proportional to the increase in TR). The *g* parameter was set to 0.27 for all participants. These values of *h* and *g* were obtained after performing a fitting with the data of Liège participants. More details about LVT and its parameters can be found in S1 File (Section B).

**Fig 2:**
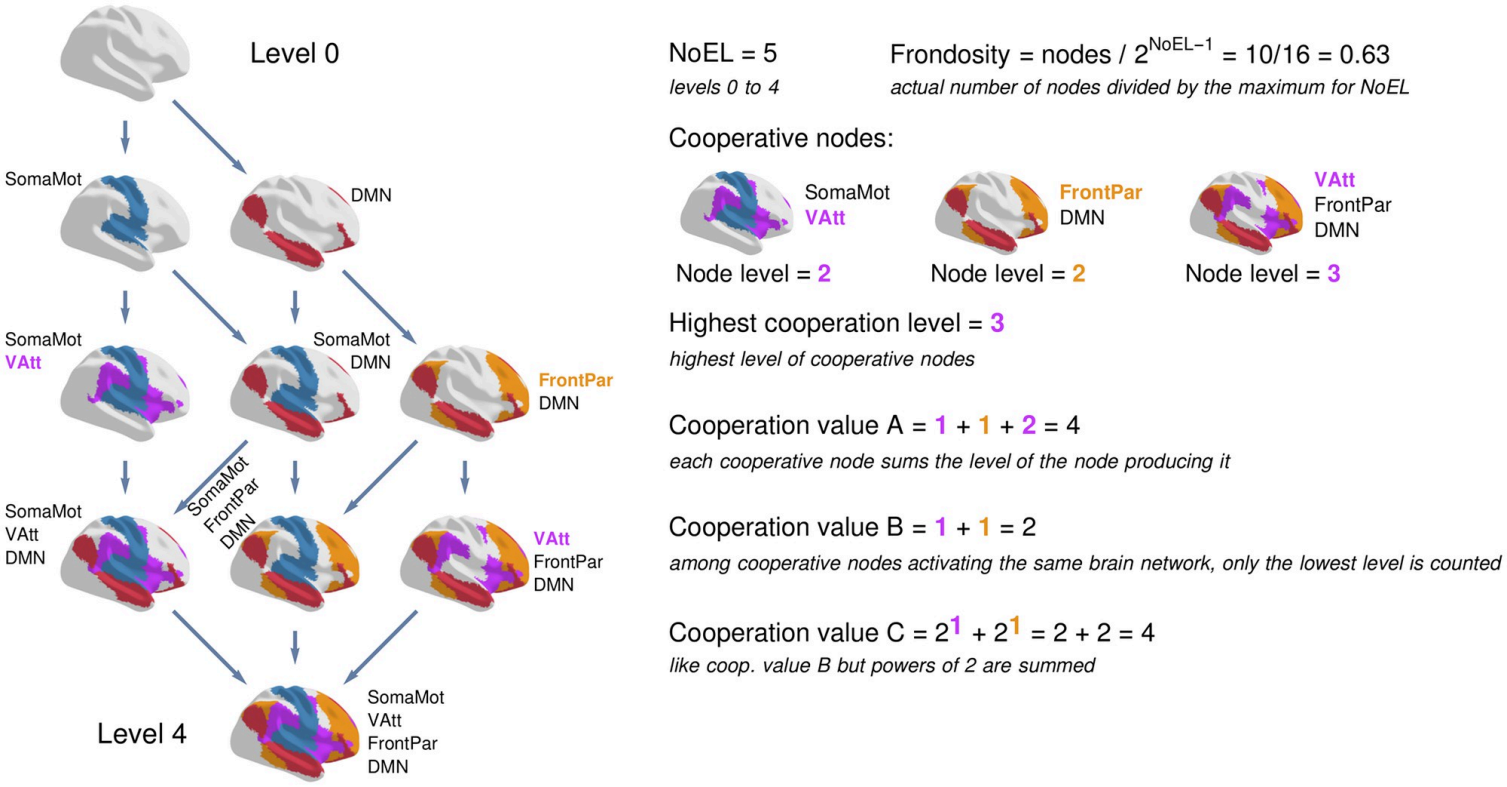
Measures of information structures. An information structure (IS) is shown on the left. It is determined by the structural connectivity matrix (fixed) and the *α*_*i*_ values (variable at each instant) for the 7 networks in Yeo’s parcellation. The nodes of the information structure correspond to stationary states of the system. Networks with a value greater than 0 are coloured. The IS can be studied through several measures. *NoEL* (number of energy levels) corresponds to the number of layers in the IS (levels 0 to 4). *Frondosity* is the ratio of nodes in the IS with respect to the maximum for its NoEL. Cooperation measures focus on cooperative nodes, which are those (except for level 1) where a network that does not appear on its own occurs in presence of other networks. The *highest cooperation level* is given by the highest level at which a cooperative node appears. Cooperation values consider cooperative nodes in different ways. *Cooperation value A* counts all cooperative nodes, adding in each case the level of the node from which it proceeds. *Cooperation value B* considers only one level (the lowest) for each new network, and *cooperation value C* operates in the same way but by adding powers of 2. Other used measures are *criticality* and *synchronicity*. *Criticality* measures how close is the globally stable node to a bifurcation point that modifies which are its active and inactive networks. Finally, *synchronicity* measures the ratio of IS of a subject (across time) in which all networks appear in the same state (active or inactive) in the globally stable solution.

Once the LVT returns the *α*_*i*_ values at each instant, the corresponding IS can be computed. By solving $u_{i}^{\prime }=0$, all solutions $\left(u_{1}^{⋆},\ldots ,u_{n}^{⋆}\right)$ are the IS stationary points. Fig 2 shows an example of the calculation of the used measures for a specific IS. In summary, the first of them, *Number of Energy Levels* (NoEL), measures the levels in the IS, which is equal to the number of *u*_*i*_ > 0 in the GASS plus one (in the example of Fig 2, there are 4 networks in the GASS, so NoEL is equal to 5). In cooperative LV systems, the set of *u*_*i*_ with a value greater than 0 in the GASS is the union of the sets of *u*_*i*_ > 0 in all stationary points of the IS. So, an IS with NoEL equal to *n* cannot contain more than 2^*n*−1^ stationary points. Then, *Frondosity* is defined as the proportion of nodes in the IS with respect to this maximum (in the example of Fig 2, it is 10/2^4^). Once the *γ* parameters of the LV equations (representing the connectivity between brain networks) are fixed, the IS depends just on the *α* parameters. A perturbation in a single *α* parameter may only affect to the precise value of the *u*_*i*_ variables in the IS nodes or may change the shape of the IS, that is, the set of stationary points and trajectories linking them. *Criticality* indicates how close is the IS to a phase transition, by looking at the *α*_*i*_ that is closest to a bifurcation that changes the shape of the IS. The smaller the criticality value, the closer the system is to a bifurcation point. *Cooperation values* (a vector of three components) measure different aspects of the interactions between nodes of the dynamical system enabling the presence (value greater than 0) of one of them in some level of the IS thanks to the presence of some others in previous levels [17]. In the example of Fig 2, only two brain networks, Somatomotor and DMN, are present at level 1, that is, these are the only networks that exist in the IS by their own. Other networks appear in the IS thanks to cooperation. For example, Visual Attention (VAtt) appears together with Somatomotor, or Frontoparietal with DMN. The nodes of the IS in which this happens are called cooperation nodes. As Fig 2 shows, a network may appear in several cooperation nodes (it is the case of Vatt, appearing in two different cooperation nodes). Cooperation measures count these nodes in different ways. The *Highest cooperation level* is the maximum level of the IS in which cooperation takes place (in the example, 3). Cooperative value A counts all cooperative nodes and cooperative value B only the lowest level in which each network appears. The difference between both values produces that A is more sensible to all interactions between networks and B is only affected by the most relevant for the *α* parameters producing the IS. Finally, cooperative value C proceeds like B but giving more relevance to the networks first appearing in higher levels of the IS. All of the so far introduced measures look at a single IS. In contrast, *Synchronicity* looks at a sequence of IS across time. It is the ratio of IS having either maximum or minimum NoEL value (all nodes of the system are together present or absent in the IS global stable point). S1 File contains more details about these measures and their computation.

Given that the *γ* parameters are kept constant (SC connectivity) and the *α* values change in time, the resulting system (for each subject) is a changing (or non-stationary) attractor landscape like that in Fig 1B. It is possible to look at the changes on the attractor governing the system behaviour by looking at the variability of the IS measures.

**Fig 3:**
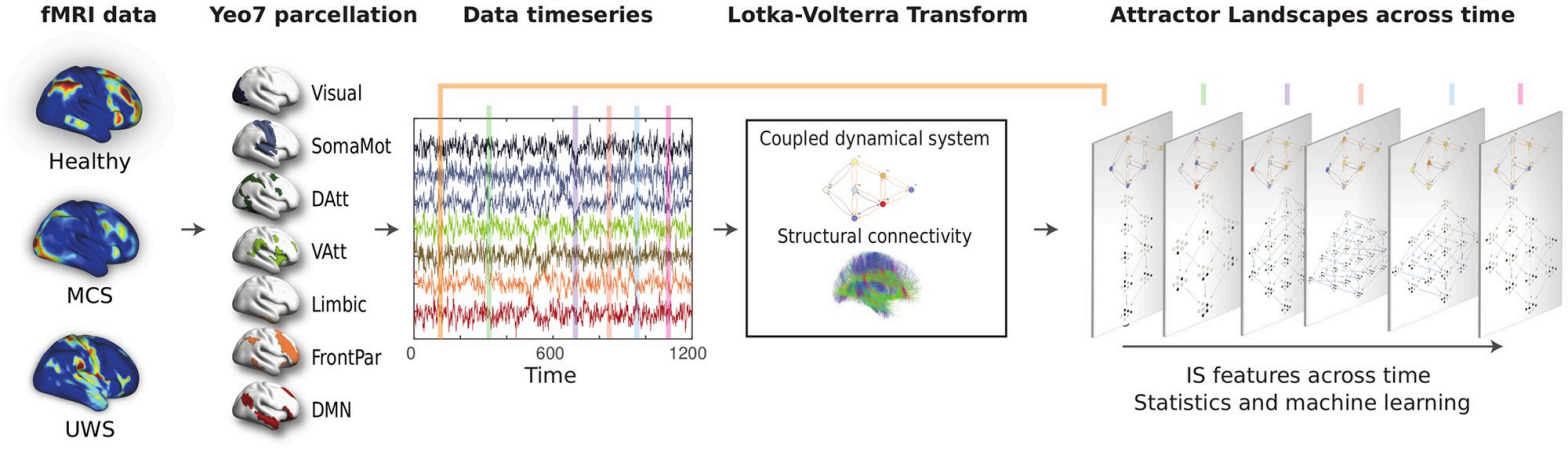
Measuring the changes in the IS underlying brain activity. The brain activity registered through fMRI is mapped into seven brain networks using Yeo’s 7-network parcellation. For each subject, given the timeseries of the activity and the structural connectivity matrix, Lotka-Volterra Transform produces the *α*_*i*_ values at each instant. This allows the study of the Information Structures evolution in time. Machine learning is used to train classifiers identifying the state of each subject.

The analysis is depicted in Fig 3. We have applied the above methodology to data corresponding to 147 participants (81 from Liège and 66 from Paris) including 49 healthy controls (HC, 36 from Liège and 13 from Paris), 63 in minimally conscious state (MCS, 31 from Liège and 32 from Paris) and 35 in unresponsive wakefulness syndrome (UWS, 14 from Liège and 21 from Paris). These datasets have been previously described in [18, 19]. The diagnosis was obtained using CRS-R by trained clinicians, with some differences between both datasets. In case of Liège patients, the diagnosis was confirmed with Positron Emission Tomography (PET), to avoid misclassification of MCS* (behavioural UWS patients presenting brain activity similar to MCS, see [20, 21]). Details on data acquisition and preprocessing are in S1 File.

All IS measures except synchronicity were calculated at each time point and the mean (or median, in the case of criticality, which is not skewed by a small proportion of outliers) and standard deviation of each measure was calculated for each subject. The different groups (HC/DOC and MCS/UWS) were compared using the Wilcoxon Signed-Rank test and then used as the input for machine learning classifiers. Our main interest in machine learning is not the classifiers themselves but to determine which IS measures are most sensitive to the state of consciousness. Some of the most important tasks when applying machine learning to a classification problem are the selection of the most relevant measures of the subjects to be classified, the choice of the learning algorithm and the optimisation of its parameters (the relevance of parameter optimisation to distinguish conscious from unconscious subjects is highlighted in [22]). There are software packages automatising these tasks (automated machine learning). A search is conducted through several learning algorithms to obtain the classifier performing the best according to certain validation criteria. The tool we used for it is Auto-Weka [23], an automatic model selection and hyperparameter optimization tool. It performs a search through the classifiers available in Weka (Waikato Environment for Knowledge Analysis), a software containing a collection algorithms for data analysis and machine learning [24]. The criterion that Auto-Weka used to select the models was performance using leave-one-out cross-validation. This strategy trains the classifier as many times (folds) as subjects are in the dataset (98 for MCS/UWS classification and 147 for HC/DOC), leaving one of them out of the training set for validation. At each fold, Weka selects the most relevant measures (feature selection) to train the classifier and the training is validated on the patient that was left out. Auto-Weka performs a search through all the classification and regression algorithms implemented in Weka by selecting the most relevant features and optimising the parameters of each classifier.

## Results

We aim to determine whether IS measures (Fig 2) provide markers of the different states of consciousness. The first column of Table 1 contains all parameters computed for each subject. In most cases, we use the mean and standard deviation of each IS measure across time. Our hypothesis is that the distributions of these measures change among the different groups of participants (HC/DOC and MCS/UWS) and that they allow to determine with a significant accuracy the state of consciousness of individual subjects. To test this hypothesis we use the Wilcoxon signed-rank test comparing the distribution of each parameter (first column of Table 1) for the different groups. The null hypothesis is that the distribution of the IS measures is similar (equal median) in the different groups. Table 1 shows the *p*-values of the comparisons between groups for centrality (mean or median) and dispersion (standard deviation) of IS measures. Low *p*-values (lower than a certain significance level *α*) ensure that the measures are detecting relevant differences in the distributions (with a probability *p* ≥ 1 − *α*). For a single measure, a typical significance level is *α* = 0.05. This threshold may be corrected for multiple comparisons given that, for *n* tests, the probability that all null hypotheses are false is (1 − *α*)^*n*^, which exponentially decreases. A classic solution to correct this is to reduce the value of *α*. The nine parameters with high %f values in Table 1 are the most relevant features for the machine learning classifiers (see Table 2) distinguishing between HC/DOC (six of them) and MCS/UWS (five). For these nine measures the Bonferroni correction lowers the significance threshold to *α* = 0.05/9 ≃ 0.0055. Most of the *p*-values for these features are less than it, so detecting relevant differences between groups.

**Table 1:** Wilcoxon Signed-Rank test for different IS measures.

		HC / DOC				MCS / UWS			
		All	Liège	Paris	%f	All	Liège	Paris	%f
NoEL	*μ*	< 10^−10^	< 10^−6^	< 10^−3^	100	< 10^−4^	0.003	0.002	0
	*σ*	< 10^−8^	< 10^−5^	0.002	0	< 10^−4^	0.004	0.004	0
Frondosity	*μ*	< 10^−13^	< 10^−11^	0.005	95	< 10^−6^	< 10^−3^	< 10^−3^	0
	*σ*	< 10^−12^	< 10^−10^	0.011	1	< 10^−6^	< 10^−3^	< 10^−3^	1
Criticality	*M*	< 10^−13^	< 10^−10^	< 10^−3^	100	< 10^−5^	< 10^−3^	0.001	94
	*σ*	0.144	0.134	0.393	0	< 10^−3^	0.003	0.010	1
Synchronicity		< 10^−13^	< 10^−9^	< 10^−4^	96	< 10^−4^	0.002	0.002	0
Coop. Level	*μ*	< 10^−11^	< 10^−10^	0.005	0	< 10^−6^	< 10^−4^	0.001	0
	*σ*	< 10^−10^	< 10^−10^	0.025	0	< 10^−6^	< 10^−4^	< 10^−3^	1
Coop. Val A	*μ*	< 10^−9^	< 10^−7^	0.010	1	< 10^−6^	< 10^−4^	< 10^−3^	100
	*σ*	< 10^−4^	0.001	0.025	0	< 10^−5^	< 10^−3^	0.003	100
Coop. Val B	*μ*	< 10^−12^	< 10^−10^	0.004	100	< 10^−6^	< 10^−4^	< 10^−3^	0
	*σ*	< 10^−10^	< 10^−10^	0.051	0	< 10^−6^	< 10^−3^	< 10^−3^	99
Coop. Val C	*μ*	< 10^−11^	< 10^−10^	0.024	1	< 10^−6^	< 10^−4^	< 10^−3^	0
	*σ*	< 10^−9^	< 10^−8^	0.066	94	< 10^−6^	< 10^−3^	< 10^−3^	82

The first column indicates the parameters computed for each subject, which in general are the mean (*μ*) and standard deviation (*σ*) of each IS measure across time. In the case of criticality, the median (*M*) is used instead of *μ*, for being more stable. The synchronicity value is by itself an average of all the IS of each subject. The Wilcoxon test compares the distributions corresponding to two different groups (HC/DOC and MCS/UWS) for each parameter. Each subject brings one value to these distributions, usually the *μ* or *σ* of an IS measure. Low Wilcoxon test *p*-values ensure relevant differences in the distributions. Machine learning is used to search for classifiers discriminating between HC/DOC and MCS/UWS. The performance of the resulting classifiers was evaluated by using leave-one-out cross-validation (Table 2). This requires to train the classifier as many times (folds) as samples are in the dataset, leaving each time one out for validation. The classifier determines which features provide more information within the training set and uses them to classify the validation sample. The %f columns show the percentage of folds using each measure in the best found classifiers. It is an indicator of the relevance of each parameter to identify the state of consciousness.

**Table 2:** Performance of the classifiers.

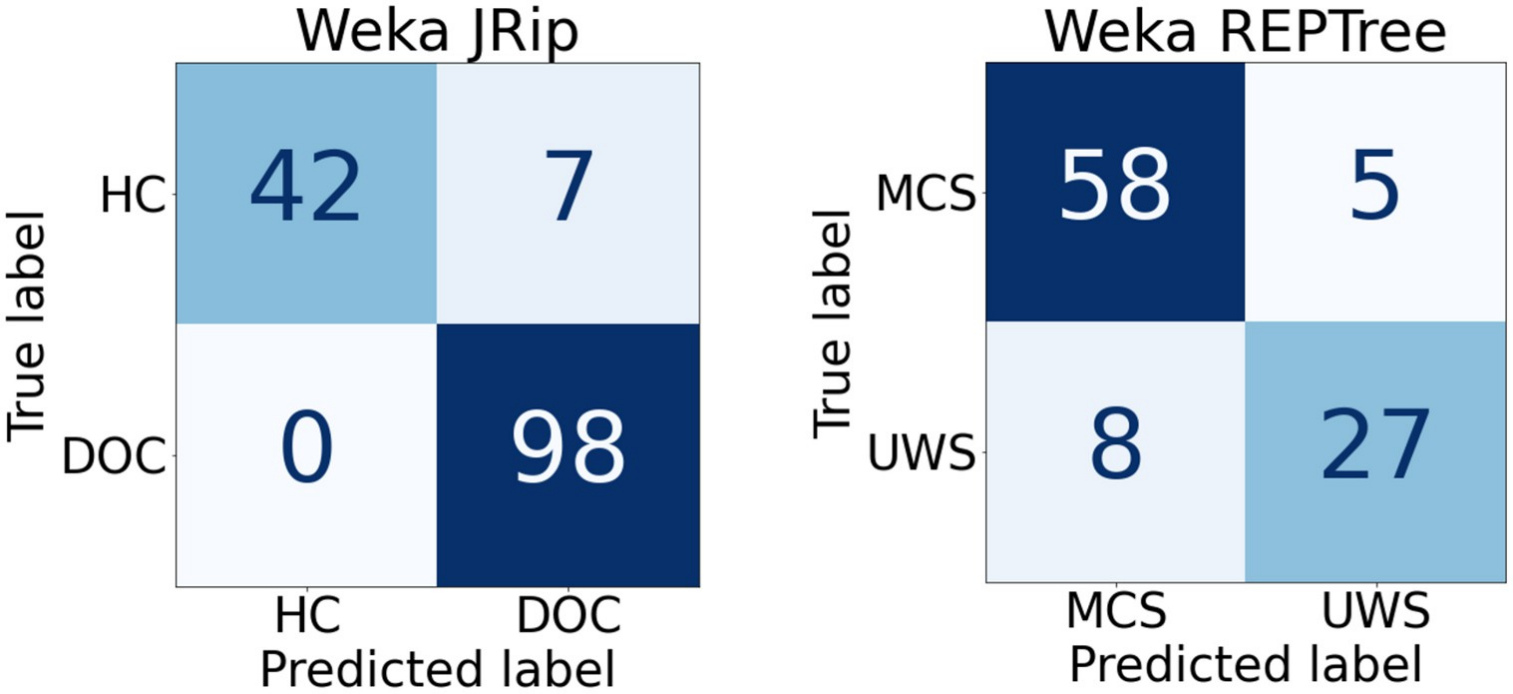									
**Classifier**	**Class**	**TP Rate**	**FP Rate**	**Precision**	**Recall**	**F-Measure**	**MCC**	**ROC Area**	**PRC Area**
JRip	HC	0.857	0.000	1.000	0.857	0.923	0.894	0.929	0.905
	DOC	1.000	0.143	0.933	1.000	0.966	0.894	0.929	0.933
Weighted Avg.		0.952	0.095	0.956	0.952	0.951	0.894	0.929	0.924
REPTree	MCS	0.921	0.229	0.879	0.921	0.899	0.707	0.938	0.953
	UWS	0.771	0.079	0.844	0.771	0.806	0.707	0.938	0.881
Weighted Avg.		0.867	0.175	0.866	0.867	0.866	0.707	0.938	0.927

Confusion matrices describing the performance of the Weka classifiers for HC/DOC (left) and MCS/UWS (right). Classifiers are trained with the 15 measures in Table 1 and validated using leave-one-out cross-validation. This strategy trains the classifier as many times (folds) as subjects are in the dataset, leaving one of them out of the training for validation. At each fold, Weka selects the most relevant measures to train the classifier and the training is validated with the patient that was left out. Table 1 (column %f) shows the percentage of folds in which each of the 15 measures was selected for training. The resulting classifiers (JRip and REPTree) were found by Auto-Weka, an automated machine learning tool. Several performance metrics are shown in the bottom table for both classifiers, for each of the classes and the weighted average. The true possitive (TP) rate (also called sensitivity) is the probability that a subject belonging to the class is correctly identified. The false possitive (FP) rate is the probability that a subject not belonging to the class will be misclassified. Precision and recall are complementary accuracy measures. Precision is the proportion of positive predictions that actually belong to the class. Recall is the ratio of positive predictions to total positive cases in the dataset. The F-measure is an accuracy measure that is calculated as the harmonic mean of precision and recall. The Matthews Correlation Coefficient (MCC) is a correlation (between -1 and 1) between the observed and predicted values. Finally the areas under the ROC (Receiver Operating Characteristic) curve and PRC (Precision-Recall Curve) are widely used measures of classifier performance. These areas are 1 for perfect classifications and classifiers with a value above 0.9 are considered to have an outstanding discrimination.

### NoEL, frondosity and criticality measures

**Fig 4:**
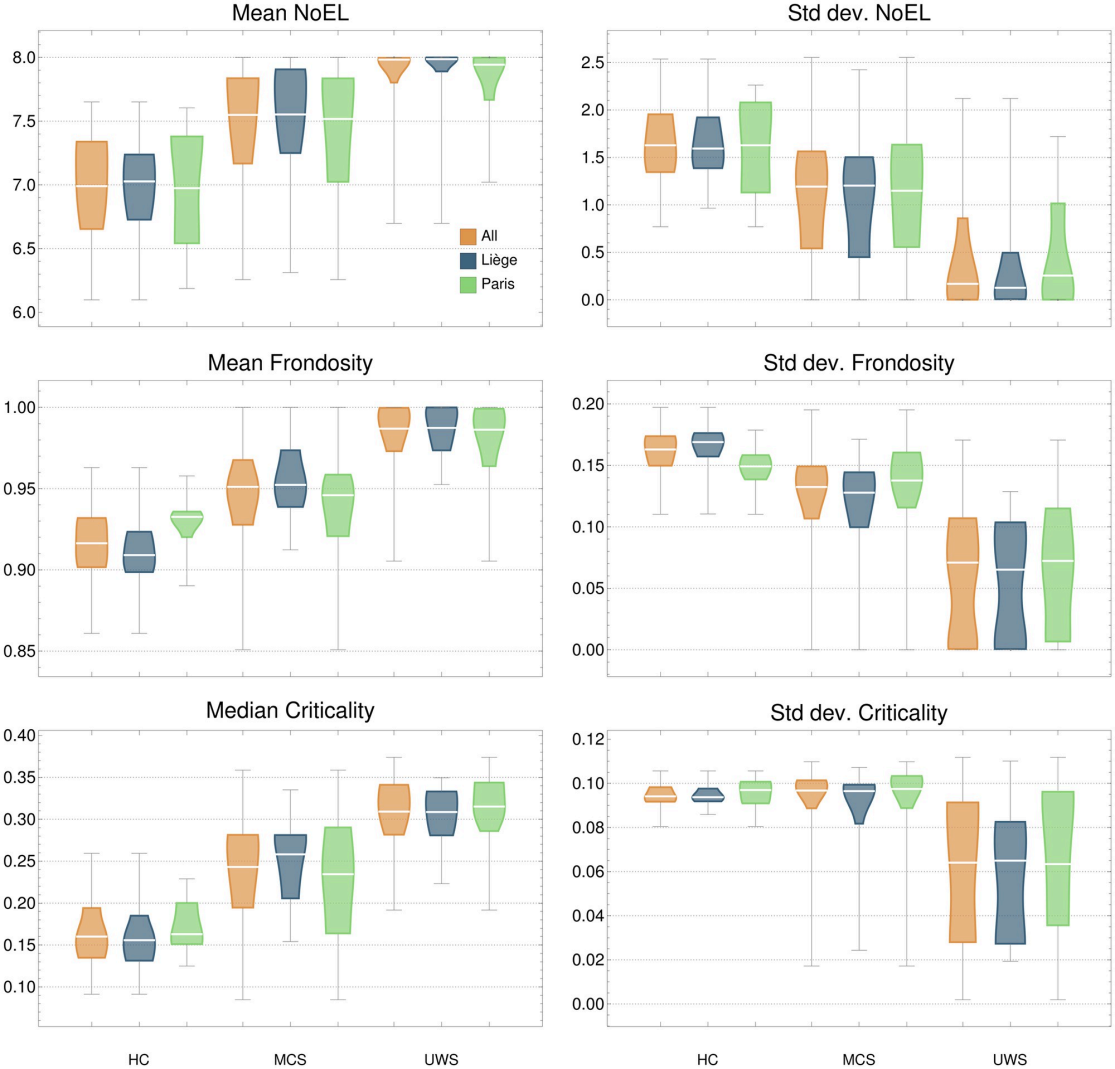
Distributions of NoEL, frondosity and criticality. Each subject brings one value to the distributions (the mean or standard deviation of the corresponding measure across time). Standard deviation of NoEL can be considered a measure of metastability, since it reflects the tendency of the system to change the attractors. Frondosity is related to integration: IS with low frondosity exhibit greater cooperative interactions between nodes (see the cooperation measures on Fig 6). Mean frondosity values very close to 1 (like those of UWS patients) denote a lack of integration in brain activity, since maximum frondosity coincides with the absence of cooperative nodes. Criticality indicates how close is the system to a major change on the attractor shape (affecting the GASS). The lower the criticality, the closer the system is to a bifurcation point and the easier it is for the IS to undergo large changes. The median criticality distributions show that the IS of healthy subjects move closer to bifurcation points.

**Fig 5:**
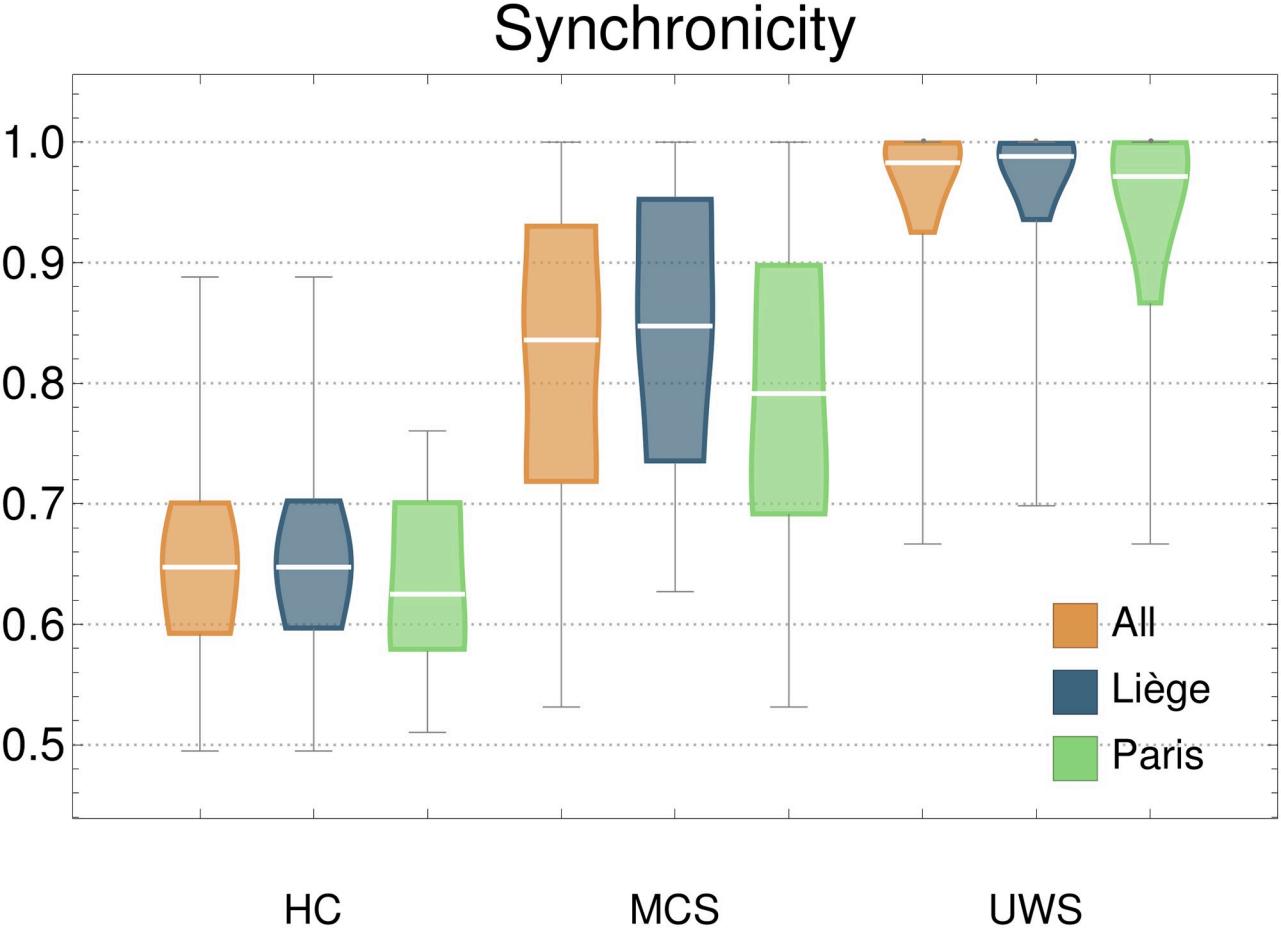
Distributions of synchronicity. This measure is not applied to individual IS. It is the ratio of IS having the globally stable point with all nodes in the same state (either 0 or greater than 0). That is, the extreme values of NoEL. Subjects with low Synchronicity have information structures with more differentiated behaviour of the nodes. UWS patients have synchronicity values very close to 1, denoting undifferentiated brain activity.

Figs 4 and 5 show the distributions corresponding to NoEL, frondosity, criticality and synchronicity for the subjects in the three groups. Together with the distributions for all subjects in the same state (All), Liège and Paris participants are shown separately to highlight the similarity between groups of subjects sharing the same state of consciousness.

Recall that frondosity measures the ratio of nodes in the IS with respect to a maximum depending on its NoEL. When frondosity is 1 it means that all the networks appearing in the GASS occur in all possible combinations in different nodes of the IS. Specifically, all networks that are in the GASS appear alone at the first level of the IS. This denotes an absence of integration between the different networks. Thus, the higher frondosity in DOC patients may be indicative of an absence of integration. This coincides with other criteria for the measure of integration [6, 25]. The variability of frondosity denotes a richer IS landscape, with interactions between networks changing over time. It can be interpreted as a measure of complexity, so that the greater functional complexity would correspond again to the control group and the lower to the unresponsive group. NoEL and frondosity are complementary metrics, as NoEL measures how many layers the IS has and frondosity measures how populated are these layers. The combination of high values of both measures that occurs in UWS patients (together with a NoEL variability close to 0) implies triviality of the IS, which continually produce the same attractor in which all networks are equally present (see Fig 5). The implication of having a single attractor over time is that the brain activity can be understood as a stationary system, whose structure remains stable and possibly oriented toward a single point of attraction. The variability in attractors (higher standard deviation of NoEL and frondosity) denotes a richer informational landscape which is not stationary. It can be considered a measure of metastability, since it reflects the tendency of the brain to move around different attractors.

Criticality was defined above as a measure of the distance of *α* parameters to a bifurcation point that would change the IS shape. Low criticality values correspond to informational landscapes moving close to bifurcation points, and therefore where richer dynamic activity may be expected. The comparison between the three groups of participants indicates that the control group is the one closest to bifurcation points, while the furthest is the unresponsive group, having more robust IS and hence more trivial information landscapes. It agrees with the idea that phenomenal consciousness is realized by a particular level of brain configuration guided by criticality and self-organisation [19, 26, 27]. An important feature of the criticality measure we have introduced is that it is not measured by looking at the time evolution of the system, but is a property that can be determined for each of its states. The average criticality over time is just used to stabilise the measure.

### The role of synchronicity and cooperation

Synchronicity measures the degree of differentiation within brain networks. The greater the differentiation, the lower the synchronicity. Fig 5 shows the distributions of synchronicity value. It is possible to refine the analysis to look for differentiation within a smaller set of networks. Focusing on DMN and frontoparietal, we can look at the proportion of GASS in which they appear synchronised (both belong to the GASS or not) or differentiated (only one of them is in the GASS). We have found that the average proportion of IS in which DMN and frontoparietal appear differentiated is 12.54% for HC, 5.48% for MCS and 2.81% for UWS. This agrees with the known anti-correlated activity of DMN and frontoparietal in conscious states which is lost in DOC patients [28–30]. The anti-correlation of dorsal attention network and default mode network has been also proposed to be characteristic of consciousness [31]. By looking at the GASS, the average differentiation of these networks is 16.96% for HC, 7.88% for MCS and 3.86% for UWS.

**Fig 6:**
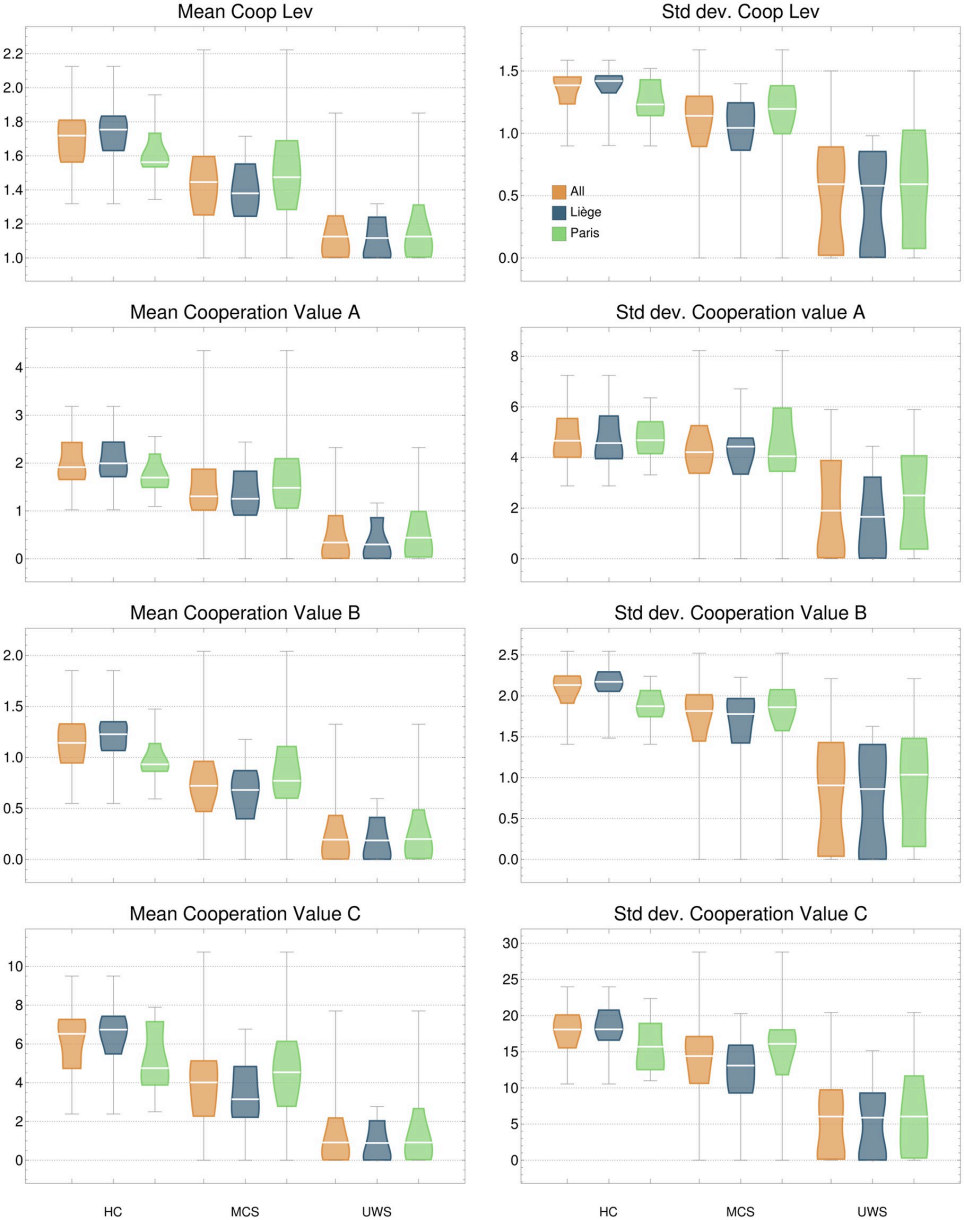
Distributions of cooperation measures. These measures consider in several ways the cooperative interactions between different networks (Fig 2). It can be observed that the higher the consciousness level, the more cooperation is in the IS (mean) and with a higher variability (standard deviation).

**Fig 7:**
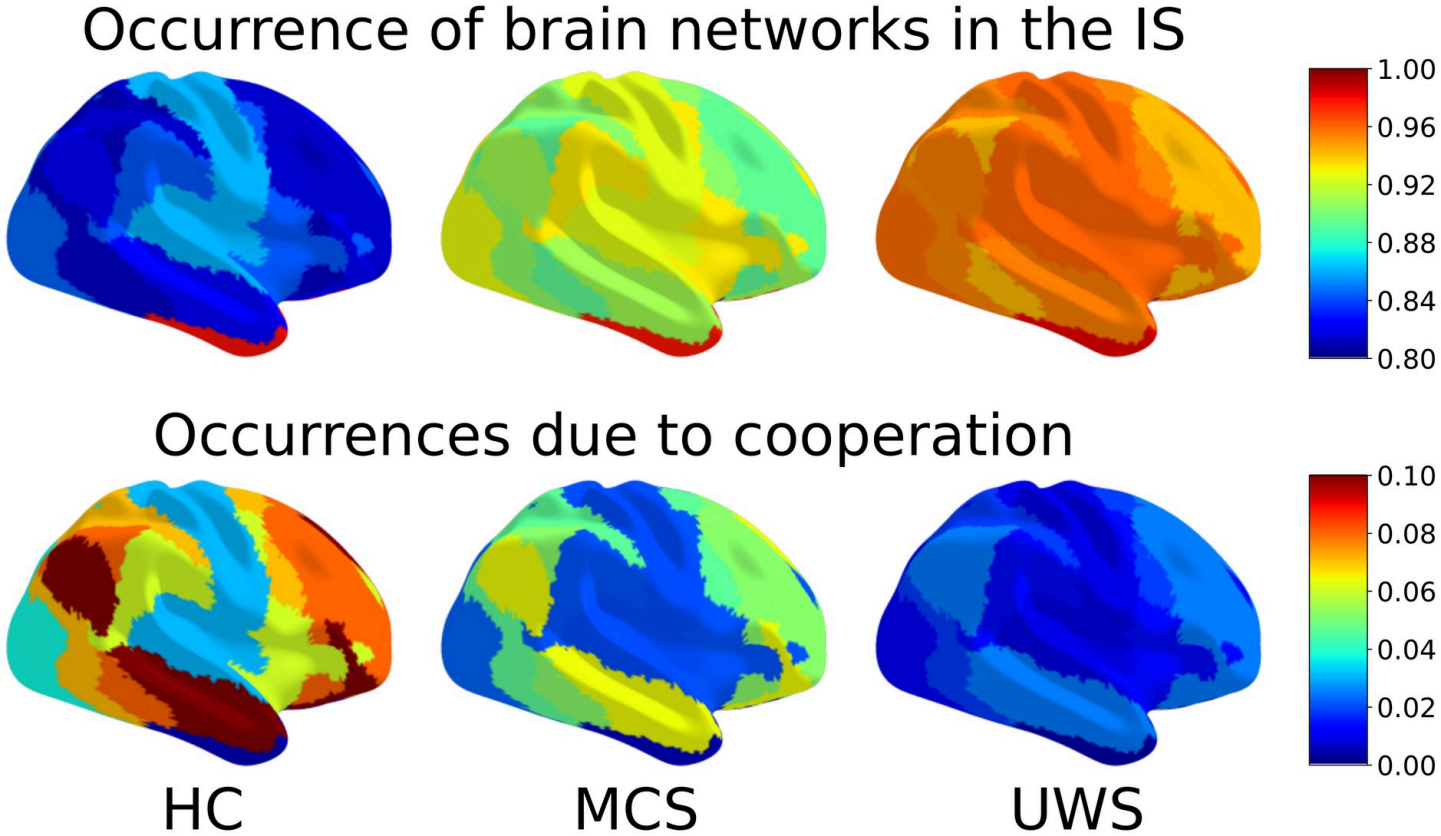
Presence of brain networks in the IS and cooperative interactions. The proportion in which each network appears in the global stable point of the IS (which is equal to appearing in any point of the IS) is shown above. Values range from 0.8 to 1. The highest values appear in UWS patients, followed by MCS. This is due to higher values of NoEL and frondosity. However, this does not imply complex behaviour in brain dynamics. The proportion of occurrences of each network in the IS due to cooperative interactions of other networks is shown below. The values are between 0 and 0.1. The highest values belong to HC (the leading group in terms of coperative measures, followed by MCS, see Fig 6). A greater differentiation between networks is observed than when looking at the global stable point alone. The highest values in all three groups correspond to DMN followed by frontoparietal.

Fig 6 shows the distributions of the mean and standard deviation of both the highest cooperation level and the different cooperation values. These measures are of particular relevance for identifying the type of brain dynamics characterising conscious states, as shown in Fig 7. In the top row, the seven networks of Yeo’s parcellation are coloured according to their presence at the globally stable point of the IS. In the bottom row, each network has been coloured according to the proportion of IS in which it appears as a result of cooperation. That is, the proportion of IS in which the network does not appear by its own (first level) but in cooperative nodes. It can be seen that healthy subjects have more cooperative interactions (mean) and higher variability across time (standard deviation). In contrast, UWS patients (and to a lesser degree also MCS) have high levels of NoEL and frondosity but the brain dynamics is almost trivial, as there is barely any cooperation among networks. The most relevant networks in terms of cooperation are DMN and frontoparietal (bottom row of Fig 7). This is also consistent with results pointing to the role of these networks in conscious activity. [32, 33].

### Machine learning classifiers

Using the IS measures in Table 1, classifiers have been trained using Weka (Table 2). Weka found a good classifier for HC/DOC based on Repeated Incremental Pruning (JRip), a propositional rule learner [34]. The classifier found for MCS/UWS is based on a Reduced Error Pruning Tree (REPTree), a classic decision tree learner [35]. Unlike other black-box machine learning methods (neural networks is the paradigmatic example), both propositional rules and decision trees offer structures that can be interpreted by humans (explainable AI). In any case, our main goal in using machine learning was to determine which IS measures are most relevant to identify the state of consciousness of a subject and to what extent it is possible to do so by looking at the shape of the attractor underlying brain activity. Table 2 (top) shows the confusion matrices of the classifiers together with several performance measures. The precision of the classifiers is 95.6% for HC/DOC and 86.6% for MCS/UWS. The areas under the ROC and PRC curves are in all cases greater than 0.92. These areas are 1 for perfect classifications and classifiers with a value above 0.9 are considered to have an outstanding discrimination [36].

## Discussion

Information structures [11, 37] have been previously used to determine the level of integrated information for different states such as wakefulness and different stages of sleep [7]. Identifying attractors in phase space and transitions between attractors may serve as useful descriptors for global brain dynamics [38], as they can be seen as a proper mathematical framework describing the space-time flow of observed dynamics. In addition, we can confront concepts with real data from human brain dynamics. The results presented in this paper are the first application of IS measures to DOC patients. Using mathematical concepts from Dynamical System Theory, our approach provides measures that discriminate subjects in different states of consciousness with a precision of 86.6% for MCS/UWS classification, which is comparable with other (f)MRI/EEG based methods in the literature [22, 32, 39].

One of the most prominent theories about consciousness is IIT, proposed by G. Tononi [40]. IIT characterises consciousness through phenomenological axioms (indicating how every conscious experience is) and postulates (establishing the characteristics that a physical system satisfying such axioms must have). IIT introduces a consciousness measure *Φ* which is calculated for a system of mechanisms in a particular state, not for the temporal evolution of the system. Our proposal is in line with this idea, since for each instant there is a different IS that can be measured. IIT proposes a structure of an informative nature, the *conceptual structure*, which determines both the level of consciousness (Φ) and the qualitative character of the conscious experiences (*qualia*). The conceptual structure is achieved by comparing probability distributions between different partitions of the system elements, in order to determine the causes and effects of a specific state. We consider that the characterisation of the IS given the state (*α* and *γ* parameters) of a dynamical system has a sounder mathematical foundation than the conceptual structure since it is supported by a solid mathematical theory that establishes that any dynamical system has an attractor of informational nature [10]. Attractors may be very different to the IS defined for cooperative LV systems but all have structured sets of stationary points and causal power. IIT axioms [40] include *existence* (“Consciousness exists: it is an undeniable aspect of reality”), *composition* (“Consciousness is compositional (structured): each experience consists of multiple aspects in various combinations”), *information* (“Consciousness is informative: each experience differs in its particular way from other possible experiences”), *integration* (“Consciousness is integrated: each experience is (strongly) irreducible to non-interdependent components”) and *exclusion* (“Consciousness is exclusive: each experience excludes all others”) that can be related to properties of the IS [11, 37]. IIT axioms have associated postulates indicating the “properties physical systems must satisfy in order to generate experience”. The *existence* postulate requires a system consisting of a set of mechanisms in one state. The existence of an IS requires also a dynamical system that at each instant is in a possible state of its phase space. It is that state which produces the specific IS. That dynamical system is composed by elementary mechanisms, as *composition* requires. Composition rules are given by the connectivity matrix and LV equations. The *information* postulate requires that each state of the system restricts its possible causes and effects to its *cause-effect repertoire*. In the same way, the IS enriches the phase space of the system with an information field [37] which contains the possible causes and effects of every state of the system. The *integration* postulate requires that the cause-effect repertoire of the system is irreducible to independent components. Cooperation measures reveal the system’s integration ability, since certain stationary points (defining the cause-effect repertoire of each state) exist because of the interaction between different mechanisms. Finally, the *exclusion* postulate introduces notions such as conceptual structure. As commented above, the notion of IS could be a candidate to determine both the quantitative (IS measures) and qualitative (shape of the information field) aspects of experience.

The calculations proposed by IIT to obtain Φ are computationally intractable for systems with a large set of elements. This is also the case with our methodology, since the IS of a system with *n* components may have up to 2^*n*^ nodes. Therefore, it is necessary to simplify the theory if we want to obtain feasible measures for such systems. The use of the seven networks of Yeo’s parcellation has allowed us to build complete IS (up to 2^7^ nodes) and to obtain numerous measures. This approach becomes intractable for large systems, but even with considerably higher sets of nodes it is possible to successfully apply some of the IS measures. Subjects awake and in several sleep stages are classified in [7] with high accuracy (92% for awake/N3 sleep) by using just the mean and standard deviation of NoEL with a brain parcellation into 90 regions [41]. Obtaining the NoEL value does not require building the whole IS but only determining the GASS. The choice of the parcellation may affect to the ability of IS measures to distinguish different states of consciousness. Selected brain areas should exhibit integration (cooperation) and differentiation (non-synchronicity) relations changing according to the level of consciousness. A larger study is to be conducted to determine which brain areas are best suited to identify the level of consciousness.

Inspired by IIT, the Perturbational Complexity Index (PCI) [42] measures the consciousness level with high accuracy. It has been validated in healthy subjects (awake, asleep, anaesthetised) and DOC patients (in different impaired consciousness states). Even when proposals have been made to define the ON and OFF states of IIT in terms of emergent properties of assemblies of neurons [43], simplifications in the theory must be assumed for practical use, like PCI does. Its value is calculated according to the temporal evolution of the brain activity evoked by Transcranial Magnetic Stimulation. According to IIT, consciousness is determined by what the system is in each state, not by how it changes through time, as PCI does. In this respect, our proposal, based on averaging IS measures over time, is more in line with IIT axioms than PCI.

The usual stationarity assumption in the use of dynamical systems for whole-brain modelling raises several practical and theoretical problems. From the practical point of view, its limitations have been pointed out [7, 44]. One of the usual practical problems is the length of the timeseries. For long data segments, it can be unrealistic to assume that all the statistics are constant throughout, while for shorter data segments, one cannot be confident that the system has explored all the states [45]. However, our measures are designed to be applied directly to non-stationary empirical data. Thanks to LVT, we obtain at each instant parameters that, when introduced in LV equations, provide information about the underneath structure of the informational flow in the human brain dynamics. We can measure each of these IS so that the length of the data segment is useful to average these measures without being a limitation. Note that LVT works like other model transforms [46] so the relevant feature of the produced IS is that it is the one corresponding to the parameters obtained by transforming with the LV model. The brain follows a dynamics very different to LV equations, but it probably has stationary points organized in a structure as any dynamical system [10], and the differences in our IS measures are plausibly due to real differences in the underneath structure of the informational flow in the human brain dynamics [47, 48].

The structure corresponding to a specific brain state depends just on the very characteristics of that state, as IIT claims, not on what it was in the past or will be in the future (although there are causal connections with past and future). On a theoretical level, assuming stationarity involves postulating a single system (and structure) underlying the whole temporal evolution of the brain. Whole-brain models often misconceive the attractor as the observed dynamics in the mechanism and not as the IS underlying such dynamics. Working with non-stationary dynamics provides a richer understanding of certain notions commonly associated with brain activity. For example, criticality is commonly associated with the ability to transit between random and ordered behaviour [49]. In our approach, this is associated with low criticality values. If *α* parameters oscillate close to bifurcation points (low value of our criticality measure), the behavior will look random (between order and chaos [50]) because small perturbations in the parameters modify the attractor structure (IS). However, a system with parameters far from bifurcation points (high criticality) will have a more homogeneous behaviour.

Another key concept related to consciousness is metastability, which is often understood to be dependent on the repertoire of brain configurations [51]. The consideration of non-stationary dynamics is by itself a source of metastability. But, in addition, the shape of each particular IS also produces metastable behaviour [17]. All points except the GASS have stability and instability directions. The GASS is a very relevant state in the dynamics of the system but by itself does not inform how and through which pathways it is reached. The other points in the IS produce a metastable dynamics in the system so that, although every point in the phase space is attracted to the GASS, the directions of attraction are influenced by all the other points of the structure [37].

Integration and differentiation are prominent features of consciousness. Unconscious states produce a decoupling between regions that causes a loss of integration [52]. Cooperation measures as integration indicators are particularly relevant. Cooperation cannot be detected by only looking at the globally stable point of the attractor, as most whole-brain models do. The relevance of cooperation in frontoparietal and default mode networks (Fig 7) can only be observed by looking underneath the globally stable solution (bottom row of the figure). This result is consistent with the relevance of these two networks for conscious activity highlighted by several studies [32, 33]. The anti-correlation observed between DMN and both frontoparietal and dorsal attention network [28–31] can be detected in our approach by a more elaborated measure of synchronicity, attending not only to the full set of networks, but to each subset, as we have shown. The GASS separates DMN from each of the other two networks to a greater extent in HC than in DOC patients.

Despite the performance of the classifiers being similar to other classifications performed with DOC patients using fMRI data, our methodology requires a validation in a larger cohort, and some measures would possibly need to be modified. In this study we have used the same SC matrix for all subjects. This has been a practical solution since individual matrices were not provided for all participants. For patients with severe brain injury the classification would possibly improve if these injuries were reflected in connectivity, since alterations within specific brain networks impairs communication among networks [53]. As we observed above, the influence of the SC matrix on LV equations is always multiplied by the activity of the respective networks. If there is no activity in a network *u*_*j*_, no matter what the connectivity *γ*_*ij*_ is, the value of *γ*_*ij*_*u*_*j*_ of the LV equations will be 0. Anyway, this does not completely solve the problem, because since we consider large regions of the brain, brain injuries can alter the connectivity without annulling *u*_*j*_ [54].

A possible new line of research is to build IS from EEG data, as well as to use functional connectivity instead of structural connectivity matrices. This would simplify the data acquisition and would turn the methodology closer to the clinical setting.

## Supporting information

S1 File Data acquisition and preprocessing. Measuring Information Structures. (PDF)
