# Supplementary material for: What lies underneath: Precise classification of brain states using time-dependent topological structure of dynamics
Source: PLoS Comput Biol. 2022 Sep 6;18(9):e1010412. doi: 10.1371/journal.pcbi.1010412 (PMC9481177; doi:10.1371/journal.pcbi.1010412)
Supplement: S1 File — (PDF) [file pcbi.1010412.s001.pdf]

What lies underneath: Precise classification of brain states using time-dependent topological structure of dynamics

Fernando Soler-Toscano, Javier A. Galadí, Anira Escrichs, Yonatan Sanz Perl, Ane López-González, Jacobo D. Sitt, Jitka Annen, Olivia Gosseries, Aurore Thibaut, Rajanikant Panda, Francisco J. Esteban, Steven Laureys, Morten L. Kringelbach, José A. Langa, Gustavo Deco

Supplementary Text

A Data acquisition and preprocessing

A.1 Participants

A.1.1 Liege

A total of 36 healthy controls (14 females, mean age $\pm$ SD, 40 $\pm$ 14 years) and 45 patients with disorders of consciousness (DOC) were included in the study based on a dataset previously described in [1, 2]. The diagnosis was conducted after at least 5 Coma Recovery Scale-Revised (CRS-R) by trained clinicians. The highest diagnosis of the level of consciousness was obtained as the conclusive diagnosis, which was also confirmed with Positron Emission Tomography (PET). Patients in MCS had a relatively preserved metabolism in the frontoparietal network, whereas patients with UWS presented a bilateral hypometabolism. Thus, we included 31 patients in MCS (9 females, mean age $\pm$ SD, 45 $\pm$ 16 years), and 14 patients in UWS (6 females, mean age $\pm$ SD, 47 $\pm$ 16 years).

A.1.2 Paris

A total of 13 healthy controls (7 females, mean age $\pm$ SD, 42.54 $\pm$ 13.64 years) and 53 patients with DOC were included in this study based on a dataset previously described in [1]. Clinical assessment and trained clinicians carried out the clinical evaluation and CRS-R scoring to determine their state of consciousness. Patients were in UWS if they showed arousal (opening their eyes) without signs of awareness (never presenting non-reflex voluntary movements). In contrast, patients were in a MCS if they presented some behaviors that could indicate awareness (i.e., visual pursuit, orientation to pain, or reproducible command following). We included 32 patients in MCS (11 females, mean age $\pm$ SD, 47.2 $\pm$ 20.76 years) and 21 patients in UWS (9 females, mean age $\pm$ SD, 39.25 $\pm$ 16.30 years).

A.2 MRI Data Acquisition

A.2.1 Liege

MRI data were acquired on a Siemens 3T Trio scanner (Siemens Medical Solutions, Erlangen, Germany). Resting-state fMRI images were acquired using a gradient-echo echo-planar imaging (EPI) sequence with 300 volumes, TR = 2000 ms, TE = 30 ms, flip angle = 78°, voxel size = $3\times3\times3$ mm, FOV = 192 mm, and 32 axial slices. The T1-weighted images were acquired with TR = 2300 ms, TE = 2.47 ms, voxel size = $1\times1\times1.2$ mm, flip angle = 9°, FOV = 256 mm, and 120 slices. DTI data were collected with 64 directions, b-value = 1,000 s/mm2, voxel size = $1.8\times1.8\times3.3$ mm, FOV = 230 mm, TR = 5,700 ms, TE = 87 ms, 128×128 voxel matrix. Approximately one non-diffusion weighted image (DWI; b = 0) per 10 diffusion-weighted images was acquired.

A.2.2 Paris

MRI data were acquired with two different acquisition protocols. In the first protocol, 21 patients and 13 healthy controls were scanned on a 3T General Electric Signa System. Resting-state fMRI images were acquired axially with a gradient-echo EPI sequence (200 volumes, 48 slices, slice thickness: 3 mm, TR=2400 ms, TE=30 ms, voxel size = $3.4375\times3.4375\times3.4375$ mm, flip angle=90°, FOV=220 mm2). The T1-weighted images were acquired in the same session (154 slices, thickness=1.2 mm, TR=7.112 ms, TE=3.084 ms, voxel size = $1\times1\times1$ mm, flip angle=15°).

In the second protocol, 32 patients were scanned on a 3T Siemens Skyra System. Resting-state fMRI images were acquired axially with a gradient-echo EPI sequence (180 volumes, 62 slices, slice thickness=2.5 mm, TR=2000 ms, TE=30 ms, voxel size: $2\times2\times2$ mm, flip angle=90°, FOV=240 mm2, multiband factor=2). The T1-weighted images were acquired in the same session (208 slices, thickness=1.2 mm, voxel size=$0.85\times0.85\times0.85$ mm, TR=1800 ms, TE=2.35 ms, flip angle=8°).

A.3 Resting-state fMRI preprocessing

Preprocessing of both resting-sate fMRI datasets was performed using MELODIC (Multivariate Exploratory Linear Optimized Decomposition into Independent Components) version 3.14 [3], which is part of FMRIB's Software Library (FSL, `http://fsl.fmrib.ox.ac.uk/fsl`). Preprocessing steps included discarding the first 5 volumes from each scan to allow for signal stabilization, motion correction using MCFLIRT [4], non-brain removal using brain extraction tool (BET) [5], spatial smoothing with 5 mm full width at half-maximum Gaussian kernel, rigid-body registration, high pass filter cutoff = 100.0s, and single-session ICA with automatic dimensionality estimation. Furthermore, we applied FIX (FMRIB's ICA-based X-noiseifier) to remove the noise components and the lesion-driven artifacts, independently, for each subject [6, 7, 8]. Specifically, we used FSLeyes in Melodic mode to manually classify the single-subject Independent Components (ICs) into "good" for signal, "bad" for lesion-driven artifacts, noise, motion, or other nuisance sources, and "unknown" for imprecise components. We classified each component by looking at the spatial map, the temporal power spectrum, and the time course. Finally, we applied FIX by using the default settings to remove the bad components and obtain a cleaned version of the fMRI data.

We used FSL tools to extract the subject-specific time series among seven resting-state networks described according to the well-known Yeo parcellation atlas: (1) Visual, (2) Somatomotor,

(3) Dorsal attention, (4) Ventral attention, (5) Limbic, (6) Frontoparietal, and (7) Default [9]. Specifically, the cleaned functional data were co-registered to the T1-weighted structural image by using FLIRT [10], the T1-weighted image was co-registered to the standard MNI space by using FLIRT (12 DOF) and FNIRT [10, 11]. The resulting transformations were concatenated and inversed, and applied to warp the Yeo atlas from MNI space to the cleaned fMRI data in native-space by using a nearest-neighbor interpolation algorithm. The averaged time series for each of the seven resting-state networks were extracted for each subject in their native-space using custom-made Matlab scripts by computing fslmaths and fslmeants. A second order Butterworth filter between 0.008 and 0.08 Hz was applied for all datasets and a piecewise-linear function was applied to the two Paris protocols to avoid systematic differences with the Liège dataset. Finally, to avoid singularities in the LVT, all values must be positive. So, the global minimum of the two datasets (all subjects, all regions) was subtracted to all data points and a small ϵ=0.01 was added.

A.4 Probabilistic Tractography analysis

The average Structural Connectivity (SC) matrix of all healthy controls in the Liège group is used for all subjects in the study, given the unavailability of the connectivity matrix of many of the DOC patients. Ideally, we would have used for each subject her connectivity matrix, but we only had them for some of the Liège participants. The choice to use a different matrix for each group (average of available SC matrices in the group) was not a good methodological decision because the IS produced were very different due to differences in the SC matrices and not in the brain activity of each subject. We therefore opted for the same matrix for all. The main limitation is that the real matrix of a patient with severe brain damage may be very different from the average matrix we used. But the LV equations multiply the connectivity parameter by the activity in each brain network, so if this is low or non-existent, the connectivity value becomes less relevant in determining the IS shape.

We generated the structural whole-brain connectivity matrix for each subject in their native diffusion space using the Yeo template as used in the resting-state fMRI data. The whole-brain matrices were computed by following the two-step procedure as specified in previous studies [12, 13, 14]. First, images in DICOM format were converted to the Neuroimaging Informatics Technology Initiative (NIfTI) format by applying dcm2nii (`www.nitrc.org/projects/dcm2nii`). The b0 image was co-registered to the T1-weighted structural image by using FLIRT [10], and the T1-weighted structural image was co-register to the standard space by using FLIRT and FNIRT [10, 11]. The transformations matrices from these steps were concatenated and inversed, and applied to warp the Yeo atlas from MNI space to the native MRI diffusion space by using a nearest-neighbor interpolation method. Second, analysis of diffusion images was performed in FSL using the processing pipeline of the FMRIB's Diffusion Toolbox (FDT) (`www.fsl.fmrib.ox.ac.uk/fsl/fslwiki/FDT`). In brief, non-brain tissues were removed by applying the Brain Extraction Tool (BET) [5], eddy current distortions and head motion were corrected by using eddy correct tool [15], and the gradient matrix was reoriented to correct for subject motion [16]. Crossing Fibres were modeled using the default BEDPOSTX parameters and the probability of multi-fibre orientations was calculated to improve the sensitivity of non-dominant fibre populations [17, 18]. Finally, Probabilistic Tractography was performed for each subject in their native MRI diffusion space using the default settings of PROBTRACKX [17, 18]. For each of the seven resting-state networks of the Yeo parcellation, the connectivity probability to each of the other six resting-state networks was estimated as the total proportion of sampled fibres in all

voxels in the network n that reach any voxel in the network p. Given that DTI does not capture fiber directionality, the SC_{np} matrix was then symmetrized by computing their transpose matrix SC_{pn} and averaging both matrices.

B Measuring Information Structures

B.1 Model Transform

Here a *model* is defined as a system of n first order ordinary differential equations with n parameters θ_i:

$$u_i'(x) = f_i(u_1, \dots, u_n; \theta_i), \quad i = 1, ..., n \tag{1}$$

where $f_i : \mathbb{R}^{n+1} \to \mathbb{R}$, and $x \in \mathbb{R}$. Given a model (1) and a differentiable function $\hat{u}$:

$$\begin{aligned}
&\hat{u} : \mathbb{R} \to \mathbb{R}^n \\
&\hat{u}(x) = (\hat{u}_1(x), ..., \hat{u}_i(x), ..., \hat{u}_n(x))^T, \text{ for all } x \in \mathbb{R} \\
&\hat{u}_i(x) \in \mathbb{R}, \text{ for all } i = 1, ..., n.
\end{aligned}$$

let us define the auxiliary functions $\hat{f}_i : \mathbb{R} \to \mathbb{R}$, $\hat{f}_i(\theta_i) = f_i(\hat{u}_1, \dots, \hat{u}_n; \theta_i)$, for all $i = 1, ..., n$. If $\hat{f}_i$ is invertible for all $i = 1, ..., n$, we define the *model transform* of $\hat{u}(x)$ for model (1) as the functions $\hat{\theta}_i(x)$ that fulfil:

$$\hat{\theta}_i(x) = \hat{f}_i^{-1}(\hat{u}_i'), \quad i = 1, ..., n. \tag{2}$$

If (1) is a model of a dynamical system we use $t \in \mathbb{R}$ (instead of $x \in \mathbb{R}$), and the parameters $\hat{\theta}_i(t)$ for any given time point $t = t_0$ are called *instantaneous parameters* in $t = t_0$.

It is easy to see that given the model (1) and given a differentiable function $\hat{u} : \mathbb{R} \to \mathbb{R}^n$, if $\hat{\theta}_i(x)$ are the model transform of $\hat{u}(x)$ for that model for $i = 1, ..., n$, then $\hat{u}(x)$ is the solution of $u_i'(x) = f_i(u_1, \dots, u_n; \hat{\theta}_i(x))$, $i = 1, ..., n$ for initial conditions $u(x_0) = \hat{u}(x_0)$ for any $x_0 \in \mathbb{R}$.

The most trivial example of model transformation is the slope of a curve at a point. The model would be the differential equation $u'(x) = m$ where m is the parameter of this model. It is obviously a "straight line model" that is trivially invertible and given a function $\hat{u} : \mathbb{R} \to \mathbb{R}$, $\hat{u}(x)$, for $x \in \mathbb{R}$ the transform is $\hat{m}(x) = \hat{u}'(x)$ which is the well-known slope $\hat{m}$ of the curve $\hat{u}(x)$ at each point x.

Another trivial example is *instantaneous velocity*. In this case the model is a uniform motion in a straight line, a trivial dynamic system, defined by the three differential equations $x_i'(t) = v_i$, $i = 1, 2, 3$. Given a function $\hat{x} : \mathbb{R} \to \mathbb{R}^3$, $\hat{x}_i(t)$, $i = 1, 2, 3$, for $t \in \mathbb{R}$, the transform is $\hat{v}(t) = \hat{x}'(t)$. Thus, in this case the instantaneous parameters are the components of instantaneous velocity.

B.2 Lotka-Volterra Transform

The Lotka-Volterra Transform (LVT) is a particular case of *model transform* (MT) when the model is given by:

$$\dot{u}_i = u_i \left(\alpha_i - u_i + g \sum_{j=1}^{n} \gamma_{ij} u_j \right), \quad i = 1, ..., n \tag{3}$$

where g is a global coupling strength parameter. From these equations we can obtain time-dependent parameters $\alpha_i(t)$ as:

$$\alpha_i(t) = \frac{\dot{u}_i(t)}{u_i(t)} + u_i(t) - g\sum_{j=1}^{n} \gamma_{ij} u_j(t), \quad i = 1, ..., n. \tag{4}$$

This equation defines the LVT. In practice, both $u(t)$ and $\dot{u}(t)$ are empirical values from BOLD signals obtained using fMRI in the form of discrete time series, γ_{ij} is the value in the SC matrix connecting regions i and j and $\dot{u}(t)$ is approximated as $(u(t+1) - u(t-1))/2h$, where h is the time parameter for the central difference approximation of the derivative. LVT transforms $u(t)$ into $\alpha(t)$. A non-stationary attractor landscape emerges when parameters α_i evolve over time. Changes in the α_i produce changes in the AL and the NoEL also changes over time. Thus, in our discrete time series at each time step k the $u_{i,k}$ data define a $\alpha_{i,k}$ column with n components ($i = 1, ..., n$), and we obtain a temporal series of T ($k = 1, ..., T$) different attractor landscapes and the corresponding T different ISs.

The global coupling strength parameter g and the parameter h are fitted to balance the information that each network i receives from its proper activity, $\dot{u}_i(t)/u_i(t) + u_i(t)$ (h affects the approximation of $\dot{u}_i(t)$) and the information coming from all the other networks, $g\sum_{j=1}^{n} \gamma_{ij} u_j(t)$ (multiplied by g). When balanced, the three terms of the LVT (4) are in the same order of magnitude. In addition, in order to get a finer fitting we optimise the differences between groups.

B.3 Global attractor and energy levels

In a n-dimensional phase space X, a dynamical system in a state u can be characterized by a family of operators indexed by time, $S(t)$, defined as $S(t)u(t_0) = u(t_0 + t)$ for $u \in X$. A given set $\mathcal{A}$ is invariant under $S(t)$ if $S(t)\mathcal{A} = \mathcal{A}$ for every t. The global attractor is a compact invariant subset of the phase space with the property that $S(t)u$ is arbitrarily close to global attractor for any u under the condition that t is sufficiently large [19].

These concepts can be illustrated using the Lotka-Volterra (LV) system, an example of dynamical system on a network:

$$\dot{u}_i = u_i\left(\alpha_i - u_i + \sum_{j\neq i}^{n} \gamma_{ij} u_j\right), \quad i = 1, ..., n, \tag{5}$$

where α_i are the growth rates and γ_{ij} the network structural connectivity. It is called cooperative Lotka-Volterra system when $\gamma_{ij} \geq 0$ for $i, j = 1, .., n$. Each stationary solution of a LV system is a unique combination of null and non-null components which can be binarized and expressed as a combination of ones and zeros (Figure 1).

The *Information Structure* (IS) of the global attractor is defined as a directed graph (see Figure 1) composed of nodes associated with the invariant subsets and links establishing their connections.

Energy levels: The global attractor is usually comprised of invariant subsets (for instance, stationary points or peridic orbits) and trajectories connecting them [19, 20]. The highest energy level is defined as formed by invariant subsets that receive no solution, i.e. unstable or source sets. Then, each successive lower level is defined including those invariant subsets that receive solutions only from the previously defined higher levels [21].

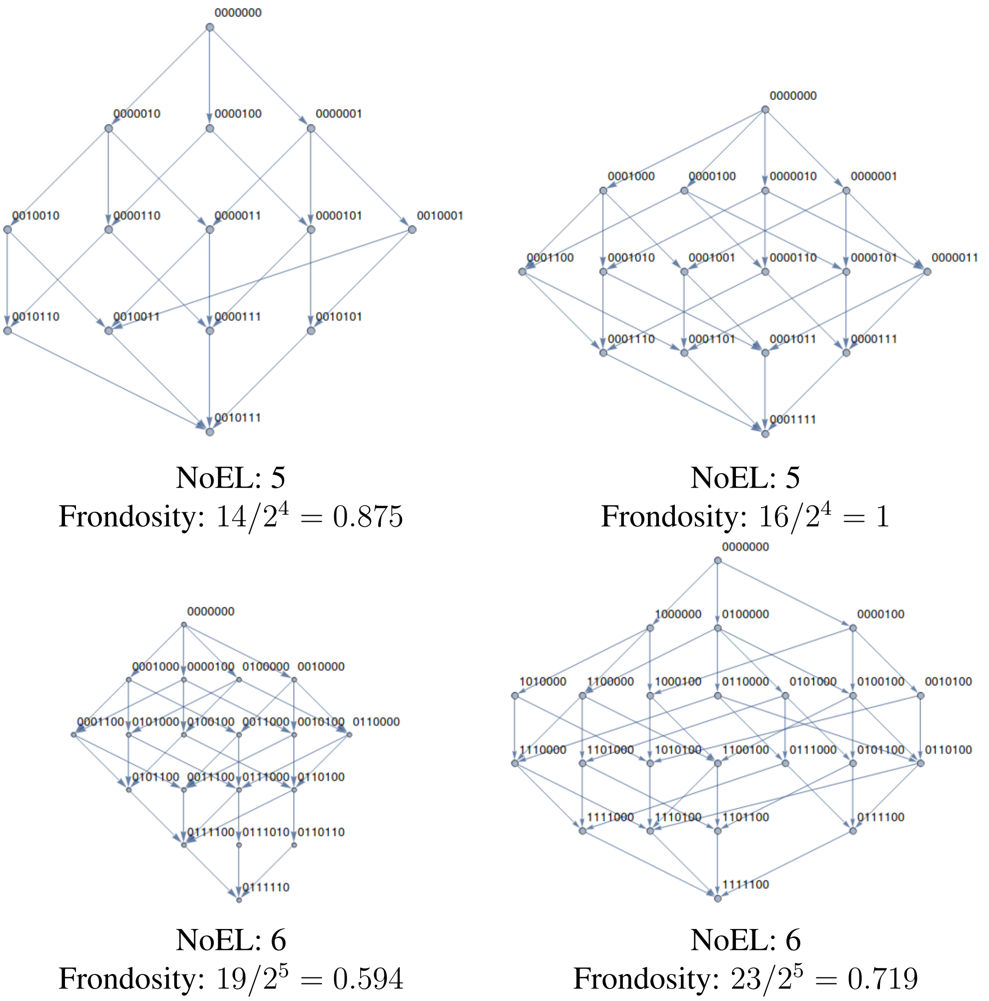

Figure 1: The Information Structure (IS) of the global attractor is defined as a directed graph composed of nodes associated with the invariant subsets and links establishing their connections. Four different Information Structures of a 7 node system are shown. Number of energy levels (NoEL) and frondosity are indicated. For LV systems each energy level is formed by stationary points with the same number of non-zero components and the only stable point is a single stationary point with only incoming solutions in the lowest level called globally asymptotically stable solution (GASS). The GASS can be said to be the point the system is attracted to, since any initial point of $\mathbb{R}^7_+$ will converge to the GASS. With $n = 7$ the attractor landscape can include up to $2^7 = 128$ stationary points.

Thus, the invariant subsets are partially ordered, i.e. they admit an order relationship in which not necessarily all the pairs of elements can be compared. The number of energy levels (NoEL) will be one of the measurement in our analysis of the attractor landscape (AL). Figure 1 displays four different examples of ISs with NoEL equal to 5 (top) and 6 (bottom).

For LV systems each energy level is formed by stationary points with the same number of non-zero components and the only stable point is a single stationary point with only incoming solutions in the lowest level called globally asymptotically stable solution (GASS). The GASS can be said to be the point the system is attracted to, since any initial point of $\mathbb{R}^n_+$ will converge to the GASS [22].

The LV model is relatively simple but, for $n = 7$ it may include a complex IS with up to 2^7 = 128 stationary points.

B.4 Frondosity

Information structures with $q+1$ energy levels (NoEL) can have up to 2^q nodes, but not all of them are always present. Frondosity is defined as the number of nodes in the IS divided by 2^q (Figure 1). This ratio is a measure of the level of integration in the system. This is because in those IS in which Frondosity is small, a stable local attractor (GASS) is reached in which ROIs are active although the nodes of the IS corresponding to many of the combinations of those ROIs do not appear in the IS. The explanation, therefore, is that the integrative interaction between different brain areas facilitates the existence of that stable attractor.

B.5 Criticality

In order to calculate the GASS a Linear Complementarity Problem (LCP) is solved [23]. Given the $\alpha \in \mathbb{R}^7$ resulting from the LVT and the matrix $M = g\Gamma - I$ of order 7, the LCP finds $(w, z) \in \mathbb{R}^{2\times 7}$, $w = (w_1, w_2, ..., w_7)^T$, $z = (z_1, ..., z_7)^T$, such that

$$w = \alpha + Mz$$
$$w \geq 0,\ z \geq 0 \ \text{ and } w_i z_i = 0 \text{ for all } i = 1, ..., 7.$$

Thus, joining w and z there are, at most, only 7 non-zero components different that are also positive and will be called r_i. Recall that each GASS of a LV system is a unique combination of null and non-null components [23]. At the points where the transition between GASS occurs there are more than 7 null components of r. Thus, one way to measure the proximity of the GASS transition is to calculate the minimum of the r_i. Previously, and since both w and z, and therefore also r, depend linearly on alpha, the vector r is normalized to make it independent of the alpha module. The minimum of the resulting $\{r_i\}$ is a measure of *criticality* as it indicates the proximity of a phase transition.

B.6 Synchronicity

The GASS divides the nodes of the system into two subsets: those equal to 0 and the rest with a value grater than 0. But there are two special kinds of IS where all nodes are in the same group. These are the IS with extreme values of NoEL (1 or 8 in the IS for the 7 networks in Yeo parcellation). Synchronicity is equal to the ratio of IS with these extreme NoEL values: all nodes

are equally active or inactive in the GASS. Lower values of synchronicity correspond to a higher differentiation in the behaviour of the nodes of the system.

B.7 Cooperation

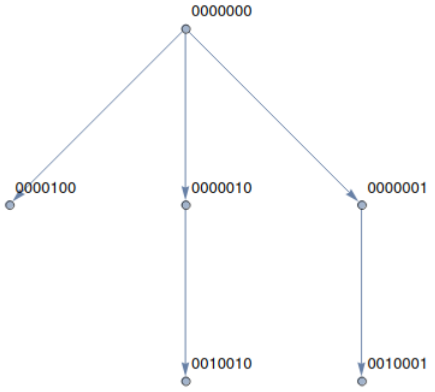

Highest coop. level: 2
Coop. value A: 2
Coop. value B: 1
Coop. value C: 2

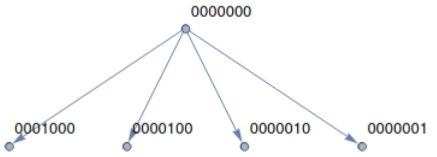

Highest coop. level: 1
Coop. value A: 0
Coop. value B: 0
Coop. value C: 0

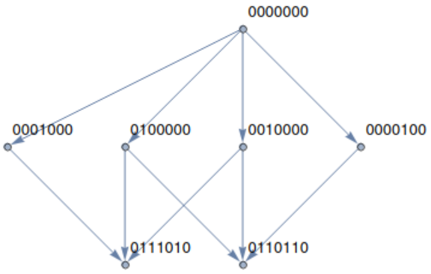

Highest coop. level: 4
Coop. value A: 6
Coop. value B: 3
Coop. value C: 8

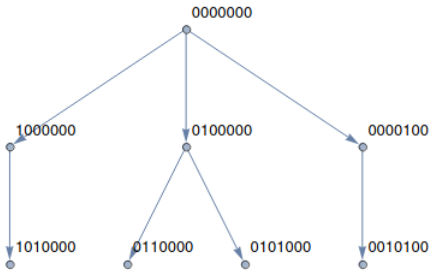

Highest coop. level: 2
Coop. value A: 4
Coop. value B: 2
Coop. value C: 4

Figure 2: Cooperative points of the Information Structures of Fig. 1.

Information Structures of cooperative Lotka-Volterra systems have some important structural properties. If two stationary points having respectively the sets A and B of nodes with a value greater than 0 belong to the same IS, then that IS contains also a stable point with nodes $A \cup B$ greater than 0. This is because of the cooperative nature of the systems. So, once a node n_i appears in a certain energy level, it will also appear in all the following levels and also in the global stable point. But we can look at the first stable point(s) where n_i appears. If n_i appears in the first energy level (like node n_6 in the top-left IS of Figure 1, which has a value greater than 0 in the solution represented by 0000010), then it is because $\alpha_i > 0$. But when n_i appears in a later energy level (like n_3 in the top-left example IS of Figure 1, which appears the first time in the solution represented by 0010010) it is because of an interaction between nodes (in the example, n_6 allows the apparition of n_3). It may happen that n_i is enabled by different nodes (in the example, n_3 is also enabled by n_7, see the node 0010001, the right-most point in the intermediate level of the IS).

We can focus on the points of an IS which contain some new apparition of a certain node, and call them *cooperative points*. Figure 2 contains the representation of the cooperative points of the four IS of Figure 1. Node 0000000 is added so that there is a common root in all cases. The top-left example corresponds to the top-left IS of Figure 1. Trivially, points in the first energy level are included because each of them contains the first occurrence of a certain node (0000100 contains the first occurrence of n_5). The only nodes in the second energy level are 0010010 and 0010001 which correspond to the apparitions of n_3 because of the cooperation of n_6 and n_7, respectively. Observe that only a minority of the points in an IS are cooperative points.

Four measures are defined by looking at the cooperative nodes of an IS. The *highest energy level* in which cooperation appears is the *highest cooperation level.* In Figure 2 the IS with the highest cooperation level is the bottom left one, which has two points 0111010 and 0110110 of level 4. By comparing figures 1 and 2, we see that the lower the frondosity, the highest the cooperation measures. The *Cooperation value A* is defined as the sum, for all cooperative points, of its level minus 1. Figure 2 indicates the cooperation values of the four examples. Recall that 0000000 is not a cooperative point. The top-left example has three points on level 1 (which sums 0 each to the first cooperation value) and two points on level 2 (which sums 1 each), so the cooperation value A is 2. The *Cooperation value B* is the sum, for each node which appears in the IS thanks to cooperation, the lower level in which appears minus one. So a node appearing due to the cooperation of several different nodes counts only once. It happens with n_3 in the top left picture of Fig. 2. It appears twice, in 0010010 and 0010001, but it only sums 1 once. In the bottom right IS, n_3 appears thrice in the second level (adding only 1) and n_4 appears once (adding 1 too). So, while cooperation value A looks at the nodes of the IS, cooperation value B looks at the apparitions of the nodes n_i of the system. *Cooperation value C* works similar to cooperation value B but adding 2^{l-1} for each node appearing the first time in level l. In the bottom left example of Fig. 2 cooperation value C is equal to 8 (2^{4-1}) because n_6 appears in level 4.

References

[1] Anira Escrichs, Yonatan Sanz-Perl, Carme Uribe, Estela Camara, Basak Türker, Nadya Pyatigorskaya, Ane López-González, Carla Pallavicini, Rajanikant Panda, Jitka Annen, Olivia Gosseries, Steven Laureys, Lionel Naccache, Jacobo Sitt, Helmut Laufs, Enzo Tagliazucchi, Morten L Kringelbach, and Gustavo Deco. Unifying turbulent dynamics framework distinguishes different brain states. *bioRxiv*, 3:2021.10.14.464380, oct 2021.

[2] Ane López-González, Rajanikant Panda, Adrián Ponce-Alvarez, Gorka Zamora-López, Anira Escrichs, Charlotte Martial, Aurore Thibaut, Olivia Gosseries, Morten L. Kringelbach, Jitka Annen, Steven Laureys, and Gustavo Deco. Loss of consciousness reduces the stability of brain hubs and the heterogeneity of brain dynamics. *Communications Biology*, 4(1), September 2021.

[3] Christian F. Beckmann and Stephen M. Smith. Probabilistic Independent Component Analysis for Functional Magnetic Resonance Imaging. *IEEE Transactions on Medical Imaging*, 23(2):137–152, feb 2004.

[4] Mark Jenkinson, Peter Bannister, Michael Brady, and Stephen Smith. Improved Optimization for the Robust and Accurate Linear Registration and Motion Correction of Brain Images. *NeuroImage*, 17(2):825–841, oct 2002.

[5] Stephen M. Smith. Fast robust automated brain extraction. *Human Brain Mapping*, 17(3):143–155, nov 2002.

[6] Gholamreza Salimi-Khorshidi, Gwenaëlle Douaud, Christian F. Beckmann, Matthew F. Glasser, Ludovica Griffanti, and Stephen M. Smith. Automatic denoising of functional MRI data: Combining independent component analysis and hierarchical fusion of classifiers. *NeuroImage*, 90:449–468, apr 2014.

[7] Ludovica Griffanti, Gwenaëlle Douaud, Janine Bijsterbosch, Stefania Evangelisti, Fidel Alfaro-Almagro, Matthew F. Glasser, Eugene P. Duff, Sean Fitzgibbon, Robert Westphal, Davide Carone, Christian F. Beckmann, and Stephen M. Smith. Hand classification of fMRI ICA noise components. *NeuroImage*, 154:188–205, jul 2017.

[8] D Carone, R Licenik, S Suri, L Griffanti, N Filippini, and J Kennedy. Impact of automated ICA-based denoising of fMRI data in acute stroke patients. *NeuroImage. Clinical*, 16:23–31, 2017.

[9] B. T. Thomas Yeo, Fenna M. Krienen, Jorge Sepulcre, Mert R. Sabuncu, Danial Lashkari, Marisa Hollinshead, Joshua L. Roffman, Jordan W. Smoller, Lilla Zöllei, Jonathan R. Polimeni, Bruce Fisch, Hesheng Liu, and Randy L. Buckner. The organization of the human cerebral cortex estimated by intrinsic functional connectivity. *Journal of Neurophysiology*, 106(3):1125–1165, sep 2011.

[10] M Jenkinson and S Smith. A global optimisation method for robust affine registration of brain images. *Medical image analysis*, 5(2):143–56, jun 2001.

[11] Jesper L R Andersson, Mark Jenkinson, and Stephen Smith. Non-linear registration aka Spatial normalisation FMRIB Technial Report TR07JA2. Technical report, FMRIB Centre, Oxford, United Kingdom, 2007.

[12] Gaolang Gong, Pedro Rosa-Neto, Felix Carbonell, Zhang J Chen, Yong He, and Alan C Evans. Age- and gender-related differences in the cortical anatomical network. *The Journal of neuroscience : the official journal of the Society for Neuroscience*, 29(50):15684–93, dec 2009.

[13] Qingjiu Cao, Ni Shu, Li An, Peng Wang, Li Sun, Ming-Rui Xia, Jin-Hui Wang, Gao-Lang Gong, Yu-Feng Zang, Yu-Feng Wang, and Yong He. Probabilistic diffusion tractography and graph theory analysis reveal abnormal white matter structural connectivity networks in drug-naive boys with attention deficit/hyperactivity disorder. *The Journal of neuroscience : the official journal of the Society for Neuroscience*, 33(26):10676–87, jun 2013.

[14] Muthuraman Muthuraman, Vinzenz Fleischer, Pierre Kolber, Felix Luessi, Frauke Zipp, and Sergiu Groppa. Structural Brain Network Characteristics Can Differentiate CIS from Early RRMS. *Frontiers in Neuroscience*, 10:14, feb 2016.

[15] Jesper L.R. Andersson and Stamatios N. Sotiropoulos. An integrated approach to correction for off-resonance effects and subject movement in diffusion MR imaging. *NeuroImage*, 125:1063–1078, jan 2016.

[16] Alexander Leemans and Derek K. Jones. The B -matrix must be rotated when correcting for subject motion in DTI data. *Magnetic Resonance in Medicine*, 61(6):1336–1349, jun 2009.

[17] T.E.J. Behrens, M.W. Woolrich, M. Jenkinson, H. Johansen-Berg, R.G. Nunes, S. Clare, P.M. Matthews, J.M. Brady, and S.M. Smith. Characterization and propagation of uncertainty in diffusion-weighted MR imaging. *Magnetic Resonance in Medicine*, 50(5):1077–1088, nov 2003.

[18] T.E.J. E J Behrens, H. Johansen Berg, S. Jbabdi, M.F.S. F S Rushworth, and M.W. W Woolrich. Probabilistic diffusion tractography with multiple fibre orientations: What can we gain? *NeuroImage*, 34(1):144–155, jan 2007.

[19] A Carvalho, JA Langa, and J Robinson. *Attractors for infinite-dimensional non-autonomous dynamical systems*. Applied Mathematical Sciences. Springer New York, 2012.

[20] M.C Bortolan, A.N. Carvalho, and J.A. Langa. *Attractors under autonomous and non-autonomous perturbation*, volume 246 of *Mathematical Surveys and Monographs*. American Mathematical Society Providence RI, 2020.

[21] ER Aragao-Costa, T Caraballo, AN Carvalho, and JA Langa. Continuity of lyapunov functions and of energy level for a generalized gradient semigroup. *Topol. Methods Nonlinear Anal.*, 39(1):57–82, 2012.

[22] Y Takeuchi and N Adachi. The existence of globally stable equilibria of ecosystems of the generalized volterra type. *Journal of Mathematical Biology*, 10(4):401–415, Dec 1980.

[23] J.A. Galadí, S. Silva Pereira, Y. Sanz Perl, M.L. Kringelbach, I. Gayte, H. Laufs, E. Tagliazucchi, J.A. Langa, and G. Deco. Capturing the non-stationarity of whole-brain dynamics underlying human brain states. *NeuroImage*, 244:118551, 2021.
